# Medical professionalism: an experimental look at physicians’ Facebook profiles

**DOI:** 10.3402/meo.v19.23149

**Published:** 2014-06-18

**Authors:** Joseph W. Clyde, Melanie M. Domenech Rodríguez, Christian Geiser

**Affiliations:** Department of Psychology, Utah State University, Logan, UT, USA

**Keywords:** professionalism, Facebook, professionalism scale, social networking

## Abstract

**Background:**

Use of social networking services (SNS) is on the rise. While many users sign in for personal purposes, it is not uncommon for professionals to connect over SNSs with clients, students, and patients.

**Methods:**

The present study used an experimental approach to examine how medical doctors’ SNS profiles impacted potential patients’ impressions of professionalism. Participants (*N*=250 students) were randomly assigned to view one of six Facebook profiles. Profiles were populated with 1) solely professional material, 2) personal material that was strictly healthy, or 3) personal material that included unhealthy behavior. Profiles portrayed a male or female physician resulting in a total of six experimental conditions. Medical professionalism was measured with the First Impressions of Medical Professionalism (FIMP) scale, specifically developed for this study.

**Results:**

There was a large and statistically significant main effect for profile type, *F*(2, 250)=54.77, *p*<0.001, $\eta_{p}^{2}=0.31$ Post hoc tests indicated that personal profiles that contained healthy behavior were rated as most professional followed by profiles with strictly professional content. Personal unhealthy profiles were rated as least professional. Additionally, female profiles consistently received higher professionalism ratings across all three profile types [*F*(1, 250)=5.04, *p*=0.026, $\eta_{p}^{2}=0.02$].

**Conclusion:**

Our results suggest that a physician's SNS profile affects a patient's perception of that physician's medical professionalism. A personal, healthy profile may augment a patient's perception of that physician's character virtues if the profile content upholds the decorum of the medical field.

Caveat emptor (‘let the buyer beware!’), a concept common in most business dealings, does not apply to members of the medical profession, in which the focus is on health and well-being rather than material goods. As the editor of *The Lancet* recently pointed out, ‘professionalism is medicine's most precious commodity … medical professionalism underpins the trust that the public has in its doctors’ (1). For a consumer of health care, the assurance of a single physician's professionalism may be essential to the image of the medical profession as a whole.

Medical professionalism has been a dynamic construct (2), with modern medical professionalism built around the physician–patient relationship. In the present study, we focus on the perceptions of medical professionalism in a sample of health care consumers, with the rationale that in the modern model of professionalism, the patient is central (3). The physician–patient relationship is most important to patients (4), and unbeknownst to the physician, this relationship may extend beyond the clinic via the Internet. In this information age, personal relationships include face-to-face contacts as well as a myriad of contacts via electronic means such as social networking services (SNS), including Facebook, Twitter, and blogs.

## Online social networking

The Internet makes it easy for consumers of health care to get in-depth information about their care providers (5). There are now more than 845 million active Facebook users, 50% of which login on any given day (6). Individuals’ profiles may be accessible to anyone, *friends* in the Facebook lingo, if privacy settings are not activated. Even if privacy settings are used, a profile may still be accessed by friends of friends, which, for the average US user, can translate to access by an additional 45,000 people. These profiles reveal not just written narratives as found in a personal email message, but a multidimensional personal profile of images, posts, groups, activities, and other private information that populate the majority of Facebook profiles.

A common belief concerning SNS profiles is that individuals present an idealized self (7). A comparison of multiple personality reports of individuals and their closest friends to their SNS profiles revealed that contrary to the idealized self-hypothesis, they found that online profiles reflected actual personality (8). The accuracy of SNS profiles as measures of personality may be because these profiles integrate personal information that reflects what is seen in personal environments. In addition, posts from friends and acquaintances are commonly found on profiles and may increase the validity of the online personality captured in a Facebook profile (7). These findings suggest that patients may be able to accurately infer the personality and characteristics of their physician by viewing the physician's Facebook profile. Moreover, these profiles may well affect patients’ perceptions of their doctors’ ability to help them in addressing their personal health care needs.

Some characteristics of a profile may be judged by broader social standards in addition to self-presentation. A recent study found that men are typically judged more positively than women when their Facebook profiles are viewed by employers (9). In videotaped doctor visits, satisfaction of a female physician was highest if the physician's behavior matched the female gender role (10). However, a more recent study found that negative stereotypes of female physicians are no longer prominent (11). To date, it is unknown whether perceptions of medical professionalism based on an SNS profile are influenced by a physician's gender.

## Physicians’ use of social networking services

In the 21st century, SNSs such as Facebook have become wells of information for anybody wanting a transparent look at another individual. At the Rouen University Hospital in France, a questionnaire was emailed to 405 residents and fellows (12). Of those who responded, a clear majority (73%) had a Facebook profile and only 61% changed the default privacy settings.

In addition to these data, two cross-sectional studies have examined the extent of Facebook use of young physicians and medical students. Of medical students (*n*=501) and residents (*n*=312) at the University of Florida, researchers found Facebook accounts for 44.5% of the students; only one-third of which were made private (13). In a cross-sectional study in New Zealand (14), out of 338 young medical graduates 65% had Facebook accounts. Of these, 37% accounts were publicly available. These profiles of young doctors revealed their sexual orientation, relationship status, religious views, and photographs of themselves drinking alcohol and intoxicated.

The current consensus is that the risks of physicians interacting with patients on SNSs outweigh the benefits (15). In primary care visits in which physician self-disclosure took place, patients reported fewer feelings of warmth, friendliness, reassurance, and comfort (16). A similar study conducted in a western New York metropolitan area also found physician self-disclosure to be unhelpful in primary care visits (17). Given potentially negative consequences of physicians’ publicly available SNS profiles, it is important to study the impact of different types of profiles on potential patients’ perceptions of medical professionalism in more detail, as was done in the present study.

## Purpose of the present study

Professional boundaries are established to protect the physician–patient relationship and to guard the profession from disrepute. SNS profiles may affect the public's perception of the physician's professionalism and of the profession as a whole. The purpose of the current study was to answer the following questions: 1) Does a physician's Facebook activity affect the public's perception of the specific physician's professionalism? 2) Does a physician's Facebook activity affect the public's perceived professionalism of the medical profession as a whole? 3) Does the level of the patients’ perceived professionalism of a physician depend on the sex of the physician?

Facebook activities were conceptualized along two dimensions: 1) professional profiles containing content strictly related to the physician's training and subsequent practice, and 2) personal profiles containing content pertaining to the physician's *nonprofessional* life. The personal dimension was further divided into two subcategories: (a) personal-healthy profiles populated with material that was strictly healthy (e.g., hiking, reading), and (b) personal-unhealthy profiles populated with material that may be considered unhealthy (e.g., overeating, sleeping-in). We hypothesized that a professional profile would lead to the highest professionalism ratings, followed by a personal-healthy profile, and finally a personal-unhealthy profile. We hypothesized that a Facebook profile in the personal-unhealthy condition would lead to low ratings of professionalism of that individual physician as well as to negative perceptions of the medical profession as a whole.

Finally, given the unclear findings regarding the influence of physician sex on ratings of professionalism, we also examined whether the level of perceived professionalism of a physician in each of the three categories (professional, personal-healthy, and personal-unhealthy) depended on the sex of the physician. It appears that physician sex is related to general impressions across areas; however, we did not have specific findings that suggested one hypothesis. Thus we investigated the role of physician sex in an exploratory manner.

## Methods

### Participants

The study was conducted at Utah State University (USU) and was approved by the institutional review board. Participants were recruited via general education courses, studentlist serves, and posts to the Facebook accounts of the researchers. Students recruited through college courses received extra credit for their participation. We excluded participants that reported having ever had a serious problem with a physician or considered suing a physician for malpractice.

### Measures

#### Screening questionnaire

In the demographics section participants were asked to provide information about age, sex, race/ethnicity, how often primary care visits took place, if there had been any major problems with their physicians, and if they had ever considered suing a physician for malpractice.

#### Professionalism: individual

We measured professionalism with the First Impressions of Medical Professionalism (FIMP) scale, developed specifically for this study. The FIMP scale was derived from a study that investigated the meaning of medical professionalism to patients, medical students, residents, and academic faculty (4). The questionnaire contains 21 statements, rated on a 7-point Likert-type scale ranging from 0 (*strongly disagree*) to 6 (*strongly agree*). To ensure content validity, all characteristics underlying medical professionalism found in the immersion/crystallization process of Wagner et al. (4) were included in the initial scale. Although Wagner and colleagues (4) identified three dimensions, we saw considerable overlap between the items of the patient relationship and the character virtues dimensions and consolidated them into a single character virtues dimension. This resulted in two dimensions of the FIMP scale: technical skills and character virtues. After preliminary item-level confirmatory factor analyses, we eliminated one item from the technical skills dimension, and three items from the character virtues dimension because of small standardized factor loadings. Both factors for the FIMP scale showed excellent composite reliabilities in the present study, McDonald's ω=0.90 for technical skills and 0.94 for character virtues.

#### Professionalism: general

The items of the FIMP scale were reconstructed from a singular format to plural (e.g., ‘This doctor is honest’ to ‘Doctors are honest’) to measure professionalism of physicians in general (FIMP-G). Participants responded on the same 7-point Likert-type scale. Both factors for the FIMP-G scale showed excellent composite reliabilities, McDonald's ω=0.88 for both technical skills and character virtues.

## Procedure

Two alumni from USU, a man, aged 32, and a woman, aged 30, played the role of the physicians (actors). The actors were chosen to match the local population, which is predominantly White American (89%) (18), and to control for possible biases in responses to the actors based on ethnic group membership. Pictures were taken of the actors in situations that matched each of the three experimental conditions. The three conditions were replicated for the two actors, resulting in six total conditions.

Three Facebook profiles were created for each actor. Screenshots of these profiles were pasted into a survey, which was administered via SurveyMonkey. Personal profiles included photos, interests, and wall posts. The professional condition only showed a profile photo and did not contain wall posts, interests, or other photos. The names of the actors, Michael and Jennifer, were the most common baby names of the 1970s (19). To avoid biases concerning sexual orientation, the actors were portrayed as heterosexual in the personal profile photos, which included the actor's spouse (a complete table of profile content is available on the web files).

A 2 (physician sex)×3 (profile type) between subjects experiment was conducted using the vignette Facebook profiles and a computer-based questionnaire to measure the perceptions of medical professionalism. Participants were randomly assigned to one of the six conditions (Table 1). After completing the demographics questionnaire, participants were shown several screenshots of the Facebook profile in their assigned condition. Participants were required to answer five simple multiple choice questions regarding the photos and wall posts such as: ‘What was the name of this physician?’ and ‘What books does this physician enjoy?’ If the participant could not identify the correct name of the physician and other details displayed on the physician's profile, it was assumed that the participant did not attend to the Facebook profile and the data from their survey responses was excluded from further analysis. After viewing the physician's profile, and completing the quiz, each participant filled out a survey to assess their perceptions of the professionalism of the physician whose Facebook profile they saw, followed by a separate survey for measuring professionalism of physicians in general.

**Table 1:** Number of participants randomly assigned to experimental conditions

	Profile type			
	
	Professional	Personal/healthy	Personal/unhealthy	
Male physician	46	40	47	*n*=133
Female physician	40	39	38	*n*=117
	*n*=86	*n*=79	*n*=85	*N*=250

## Results

In total 324 participants began the survey and 277 (85.5%) completed. Of those that completed, 27 (9.7%) were excluded due to inaccurate answers to the questions designed to determine if the participant attended to the primary stimulus. This resulted in a final sample size of 250 participants. The majority of the sample was made up of participants who were single, attended college, and lived in Utah. The demographics for all participants are summarized in Table 2.

**Table 2:** Demographics of participants who completed and met criteria for inclusion in the study

Variable	*n* (%)
Gender (n, %)	
Male	77 (30.8)
Female	173 (69.2)
Race/ethnicity (n, %)	
Asian	6 (2.4)
Black or African American	1 (0.4)
Hispanic or Latino	7 (2.8)
American Indian or Alaska Native	1 (0.4)
White or European American	221 (88.4)
Multiracial	8 (3.2)
Rather not say	6 (2.4)
Attending college (n, %)	218 (87.2)
Visits to primary care physician (n, %)	
Never	14 (5.6)
Less than once a year	124 (49.6)
One to three times a year	94 (37.6)
More than three times a year	18 (7.2)
Age (years; M±SD)	23.4±7.6
Min, Max	18, 66

To address any potential bias due to the sex of the participant, an independent-samples *t-*test was conducted to compare FIMPs between men and women in the sample. There was no significant difference in the scores for men (*M=*3.40, *SD=*0.99) and women (*M=*3.44, *SD*=0.99); *t*(286)=0.377, *p*=0.706, in physician's professionalism ratings.

**Fig. 1:**
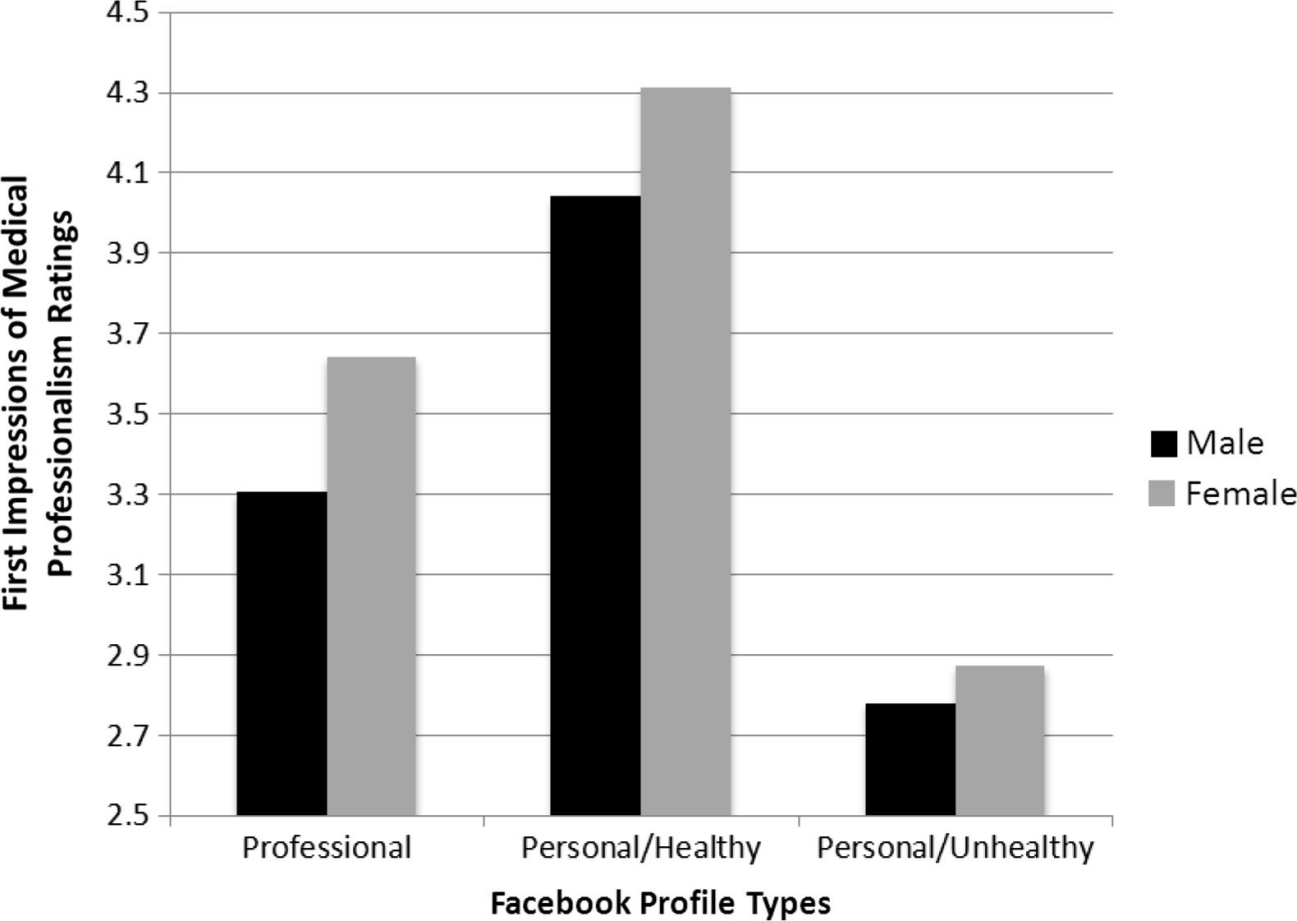
First impressions of medical professionalism ratings by profile type.

A 2 (physician sex)×3 (profile type) ANOVA was conducted with the individual physician's professionalism ratings as the outcome. There was a large and statistically significant main effect for profile type indicating a difference between profile type and the level of perceived professionalism of the profile owner (Table 3). Post hoc tests indicated that the personal-healthy condition was rated as most professional followed by the strictly professional profile. The personal-unhealthy profile was rated as least professional. There was also a statistically significant main effect for the sex of the physician indicating that male and female physicians were rated differently on their level of professionalism with the female receiving a higher rating than the male. Finally, the physician sex by profile type interaction was not significant indicating the effect of profile type did not depend on the sex of the physician. The mean rating of professionalism for each condition is given in Fig. 1.

**Table 3:** ANOVA results for FIMP ratings of individual physicians and physicians in general

	*df*	*F*	$\eta_{p}^{2}$	*p*
Individual physician				
Profile type	2	54.77	0.31	<0.001*
Physician sex	1	5.04	0.02	0.026*
Profile type/sex interaction	2	0.50	0.004	0.609
Physicians in general				
Profile type	2	0.37	0.004	0.691
Physician sex	1	0.62	0.001	0.431
Profile type/sex interaction	2	0.63	0.002	0.543

* denotes a statistically significant finding.

A 2 (physician sex)×3 (profile type) ANOVA with the physicians in general professionalism ratings as the outcome revealed that there was no significant physician sex by profile type interaction indicating the effect of profile type did not depend on the sex of the physician concerning the participants’ perceptions of professionalism for physicians in general. There was not a significant main effect for the sex of the physician indicating that there was no difference in the perceptions of professionalism of physicians in general between the male and female profiles conditions. Finally, there was no significant main effect for the profile type indicating that professionalism of physicians in general was not affected by viewing either the professional, personal-healthy, or personal-unhealthy profile of the physician actors.

## Discussion

In this study we examined 1) whether a physician's SNS profile content had an effect on potential patients’ first impressions of the profile owner's professionalism, 2) whether the SNS profile content affected impressions of professionalism of medical doctors as a whole, and 3) whether these perceptions of professionalism depended on the physician's sex. The main findings were that the personal-healthy SNS profiles were rated as more professional than both the personal-unhealthy profiles and strictly professional profiles and the female profiles were associated with higher professionalism ratings compared to the male actor profiles. Impression of professionalism of medical doctors as a whole was not affected by the physician SNS profiles.

It was no surprise that the personal-healthy conditions predicted higher perceptions of professionalism over personal-unhealthy condition. However, it was unexpected that the personal-healthy condition would be regarded as more professional than the professional profile. Upon further consideration, physician–patient attitude congruence has been linked favorably to patient satisfaction (20). The importance of personal information has also been evident in medical students’ student–teacher relationships (21). Participants in the personal-healthy conditions had sufficient information to make conclusions on whether the physician possessed a particular character trait, which matched their own personality. Participants in the strictly professional condition probably felt that they did not have sufficient information to come to a conclusion and thus may have been more conservative in their inferences on character trait matching.

These findings are consistent with a study that investigated the effects of teacher self-disclosure via Facebook on students’ perceptions (22). Student participants in the high self-disclosure condition reported higher levels of teacher credibility when compared to the low self-disclosure condition. This suggests that our findings on patients’ perceptions of a doctor's professionalism may also translate to the classroom. In the same way a strong physician–patient relationship is necessary in medical care, in medical education, the student–teacher relationship holds a fundamental role in achieving the curriculum goals (23). Knowing a little about a teacher's personality through online profiles may help the student identify with the teacher. Additionally, one of the most effective methods for teachers to transmit professional behavior is to be exemplars of professionalism in all environments – including the increasingly common online environments (24).

Another interesting finding of the present study was the higher ratings of professionalism found for the female actor's profiles compared to the male actor's profiles. Although, upon examination of the FIMP scale items, several of the character traits (e.g., caring, compassionate, ability to show emotion) are more commonly associated with women (25). These findings coincide with past research that found that female physicians in a primary care setting engage in more empathetic behavior and patient-centered practice styles (26, 27).

In the present study the perceived professionalism of physicians in general was not affected by the Facebook profile of the individual physician. One interpretation is that the acts of one physician are understood by potential patients as individual actions rather than reflecting all medical professionals. This finding may also have emerged because of the nature of the unhealthy practices. The personal-unhealthy profile contained common unhealthy practices (e.g., overeating, sunbathing, drinking, popular media entertainment). If the content of the unhealthy condition were more extreme or consistently found across multiple physicians’ profiles, this may indeed affect the image of the whole profession.

Perceived personal characteristics of a physician such as empathy have been shown to affect patient compliance (28). More recently, researchers have found that the competence of a physician is assumed unless otherwise proven wrong and therefore the personal characteristics of a physician become important (29). Given these results, future investigation of how perception of a physician's professionalism will affect patients’ adherence to medical advice, medical attention seeking, and selecting a medical professional, is warranted. Finally, another direction for future research concerns professionalism in the context of Facebook disclosures of patient information. For example, how do patients and students perceive a physician whose Facebook profile contains data on their patients? (e.g., Wall status: Today I saw a patient that was quite difficult). To date, no research has been published on patient attitudes towards their physician's online presence and it would be worthwhile to know if certain material may appear as a breach of confidentiality.

The present study contains two notable limitations: the uniformity of the participant demographics, and the use of only one man and one woman physician actors. Because the sample was mainly drawn from a university community, the study's findings may have limited generalizability. Also, because we only used one man and one woman, the main effect that physician sex had on the perception of medical professionalism could be simply due to the specific actors. It would be worthwhile to conduct an extended experiment, in which people rate the profiles of multiple physicians, so that potential specific biases can average out to determine the robustness of the effect of physician sex found in our study.

## Conclusion

As an increasingly larger portion of the world's population socially engages in the Internet (30), the frequency of problems relating to professional matters is very likely to increase as well (31). Medical professionals need to be aware of the potential benefits and dangers of participation. The present study shows that initial exposure to a physician's SNS profile may be important in forming a first impression of specific physicians. Personal unhealthy profiles that do not match the decorum of a physician are decreasing the perceived professionalism of that physician. Alternatively, personal healthy profiles may increase the perceived character virtues of that physician more so than an online profile containing strictly professional information.

We recommend that medical doctors carefully monitor their SNS profiles and if any content that may be viewed in a negative light is found, take advantage of the privacy settings. For example, physicians (and anyone) can restrict the shared content of their Facebook page by creating groups that have partial access to information. By creating a ‘patients group,’ physicians can restrict the amount of information patients can have access to without limiting the information they can post to a more personal community of friends and family. However, our results suggest that making some personal information open to the public may be beneficial as long as the content upholds the decorum of the medical field. Personal characteristics of the physician are fundamental in establishing successful physician–patient relationships and these characteristics may be more easily inferred from a multidimensional SNS profile (32). Patients seeking a doctor could potentially receive comfort by knowing a little more about the individual that will provide for their care.
